# Introducing PALETTE: an iterative method for conducting a literature search for a review in palliative care

**DOI:** 10.1186/s12904-018-0335-z

**Published:** 2018-06-02

**Authors:** Marieke Zwakman, Lisa M. Verberne, Marijke C. Kars, Lotty Hooft, Johannes J. M. van Delden, René Spijker

**Affiliations:** 1 Julius Center for Health Sciences and Primary Care, University Medical Center Utrecht, Stratenum 6.131, PO Box 85500, 3508 GA Utrecht, Netherlands; 2 Cochrane Netherlands, University Medical Center Utrecht, Utrecht, The Netherlands; 3 Medical Library, Academic Medical Center, University of Amsterdam, Amsterdam, Netherlands

**Keywords:** Palliative care, Literature search, Review, Iterative method

## Abstract

**Background:**

In the rapidly developing specialty of palliative care, literature reviews have become increasingly important to inform and improve the field. When applying widely used methods for literature reviews developed for intervention studies onto palliative care, challenges are encountered such as the heterogeneity of palliative care in practice (wide range of domains in patient characteristics, stages of illness and stakeholders), the explorative character of review questions, and the poorly defined keywords and concepts. To overcome the challenges and to provide guidance for researchers to conduct a literature search for a review in palliative care, Palliative cAre Literature rEview iTeraTive mEthod (PALLETE), a pragmatic framework, was developed. We assessed PALETTE with a detailed description.

**Methods:**

PALETTE consists of four phases; developing the review question, building the search strategy, validating the search strategy and performing the search. The framework incorporates different information retrieval techniques: contacting experts, pearl growing, citation tracking and Boolean searching in a transparent way to maximize the retrieval of literature relevant to the topic of interest. The different components and techniques are repeated until no new articles are qualified for inclusion. The phases within PALETTE are interconnected by a recurrent process of validation on ‘golden bullets’ (articles that undoubtedly should be part of the review), citation tracking and concept terminology reflecting the review question.

To give insight in the value of PALETTE, we compared PALETTE with the recommended search method for reviews of intervention studies.

**Results:**

By using PALETTE on two palliative care literature reviews, we were able to improve our review questions and search strategies. Moreover, in comparison with the recommended search for intervention reviews, the number of articles needed to be screened was decreased whereas more relevant articles were retrieved. Overall, PALETTE helped us in gaining a thorough understanding of the topic of interest and made us confident that the included studies comprehensively represented the topic.

**Conclusions:**

PALETTE is a coherent and transparent pragmatic framework to overcome the challenges of performing a literature review in palliative care. The method enables researchers to improve question development and to maximise both sensitivity and precision in their search process.

## Background

Palliative care (PC), a relatively young specialty, is growing rapidly and will continue to do so over the next decades [1, 2]. The values of PC, such as adequately controlling symptoms, alleviating the burden of patients and informal caregivers, and preventing unnecessary hospitalisations [3, 4] have been presented in an increasing number of scientific publications [5–7]. Clinical practice is preferably guided by a sufficient body of high quality evidence from research in combination with clinical expertise and patients’ preferences [8]. To inform evidence-based guidelines and protocols, the need for literature reviews in PC is pressing. Literature reviews summarise and appraise the best available evidence on a topic and are considered the highest quality of evidence for evidence-based medicine [9, 10].

Widely used methods for literature reviews are developed primarily for intervention studies and have been applied to other fields, including PC. However, there is a need for literature reviews in PC beyond those that seek to pool evidence from intervention studies. The methods used for reviews concerning evaluation of interventions may not be transferable to literature reviews on less clearly defined topics that involve different challenges [11]. One of the challenges in PC is to build review questions based on the four parts of the PICO framework (Patient-Intervention-Control-Outcome). The challenge for PC is characterised by the wide range of domains due to variations in patient characteristics, disease trajectories, stages of illness, management of treatments, and involved stakeholders, which leads to a variety of topics, such as symptom management, psychosocial care, decision-making, and health services [1, 6, 7, 12]. A developing discipline such as PC often uses explorative review questions to gain a better understanding of the topic of interest, for example: ‘How do patients with chronic heart failure experience an exercise programme to reduce illness related fatigue?’. The heterogeneity in practice and the explorative nature of the questions have hampered the use of PICO, which should be considered by a researcher when developing the review plan. Different frameworks have been developed to handle this variation, such as SPICE (Setting, Perspective, Intervention, Comparison, Evaluation) or SPIDER (Sample, Phenomenon of Interest, Design, Evaluation, Research type), but the aforementioned challenges remain [13–16].

After formulating a review question, the next stage of study identification has its own challenges. A young discipline such as PC often suffers from concepts and terms that are heterogeneous, poorly defined, indexed, or standardised, making term-based searching difficult. This is not unique for PC, as similar problems have been encountered in social sciences [11, 12, 17]. Consequently, indexing systems such as MeSH (Medical Subjects Headings, the controlled vocabulary thesaurus of MEDLINE) do not cover many key concepts within PC. Furthermore, most general bibliographical databases only publish the author written abstracts together with independently annotated indexing terms. However, relevant information for PC review questions is not always part of the original study objective or is only presented as a subtopic and not reflected in the abstract. In these cases, a perfect match search based on the elements of the review question will not be sufficient to retrieve relevant studies. Therefore, a different approach for identifying key representational features within abstracts to discover these articles needs to be employed. Taken together, poor indexing, and the heterogeneous use of terminology will result in an unbalance between specificity and sensitivity. To specify, either ineffective searches missing many relevant articles or inefficient search strategies resulting in very high numbers of search results, tens of thousands, that must be screened manually. To narrow down results in intervention studies, a component on study methodology is added to the search query. However, most research within PC cannot be answered by randomised controlled trials, [18–20] rather, it relies heavily on alternative study designs such as mixed methods and qualitative studies [12, 21]. Since the preferred study design is not always clear at the start and most research papers poorly report the applied methodology, the use of methodological search filters has been contested [22]. Although some success using filters has been reported, the broad terms used will yield low-precision results and, therefore, a high number of needed-to-screen (NNS) [22]. This phenomenon has also been seen in fields such as diagnostic accuracy [23].

Although the Boolean search query is most widely used in literature reviews, it is not the only way of retrieving studies or finding information. Other retrieval methods, including berry picking (Table 1), pearl growing (Table 1), and snowballing, have their own strengths and weaknesses. Berry picking is difficult to reproduce and lacks transparency, but has the advantage of gaining knowledge and identifying knowledge gaps with each item (berry) found. Pearl growing can help in identifying the relevant phrases and indexing terms used within the field, but is highly dependent on the composition of the initial set. Using the knowledge of peers regarding the relevance of studies, can reveal information not available in the abstract, but runs the risk of bias towards the predominant view within the field. For literature reviews, transparency and reproducibility are key features and, therefore, the Boolean logic query is so popular, as it is transparent in what it does, all elements are visible, and it is reproducible.

**Table 1:** Search techniques and analytic tools

Search techniques and analytic tools	Explanation
Berry Picking	Berry Picking is a retrieval model where obtaining evidence is not a linear path, but an iterative process where each newly identified piece of information can result in a modification of the information base required. Various techniques are used to identify the next piece of relevant information such as footnote chasing, journal browsing or database searching. Where it differs, is that information is not returned as a complete set, but in bits and pieces (the berries) informing the information base as one goes along [24].
Pearl growing	In the process of pearl growing, relevant articles to the topic of interest are identified and they enable researchers to isolate keywords and index terms on which the researchers can base their search. By using these identified keywords and index terms to build the search, the corpus of relevant articles will grow. This process is repeated for all initial papers and newly identified relevant papers for either a predetermined number of times or until no new relevant papers are identified [10, 25].
Citation tracking	For citation tracking, researchers search for all articles which were cited by relevant articles (backward citation tracking) and for all articles which cite the relevant articles (forward citation tracking). Every found reference has been deemed relevant after careful consideration by the researchers. As such, researchers make use of the ‘knowledgeable crowd’. That is, a corpus can grow through citation tracking based on the knowledge present within the literature by peers based on their knowledge and judgement of the content of the full article [11, 22].
‘Golden bullets’	‘Golden bullets’ are articles that align with the inclusion criteria for the systematic literature review and, therefore, undoubtedly should be part of the review. The ‘golden bullets’ are used for feature extraction to inform the Boolean search strategy. Furthermore, the ‘golden bullets’ are used in the validation test of the search. During the validation, the reviewer is checking whether the ‘golden bullets’ are included in the outcome of the search, ensuring a suitable search strategy to identify relevant studies.
Software	During the iterative method, some text analysis tools can be used. For instance, during the analysis of the ‘golden bullets’ the analysis tools present in Eppi reviewer [33] can be used. A possible tool for word frequency is the TF*IDF option, which helps to identify relevant terms and PubReMiner (http://hgserver2.amc.nl/cgi-bin/miner/miner2.cgi). PubReMiner is an online resource to which PubMed search queries can be submitted to produce a list and frequency counts for all keywords (subheadings, title-words etc.) and MeSH-terms associated with the articles in that query. Swift-review is an interactive workbench that provides numerous tools to assist with literature prioritization. The software utilizes recently developed statistical modelling and machine learning methods that allow users to identify over-represented topics within the literature corpus and to rank-order titles and abstracts for manual screening [34]. To identify multi-word phrases, n-grams, the Termine tool can be used [35]. For identifying concepts within the ‘golden bullet’ set, it can be helpful to use cluster analysis [36] within Eppi reviewer, which is an application of the Lingo3 engine. Results of the search can be loaded in Endnote X7 (or any other suitable program for managing references) for deduplication. In the absence of Eppi reviewer a plethora of tools is available on the web like voyant-tools (https://voyant-tools.org) for term frequency analysis, termine on the web for n-grams (http://www.nactem.ac.uk/software/termine/) and vos-viewer for cluster analysis (http://www.vosviewer.com). For more information see http://systematicreviewtools.com.

To address the aforementioned issues, there is a need to combine several of the existing retrieval methods in a logical way to ensure transparency and provide guidance for researchers. To reflect the more iterative nature of searching for PC studies, we developed a pragmatic framework, Palliative cAre Literature rEview iTeraTive mEthod (PALETTE), to guide the fine-tuning of the review question, performing a literature search, and applying screening eligibility criteria. By introducing intermediate validation steps, the reasoning for going from one phase to the next within the framework becomes visible which increases the transparency. It is the combination of these iterative steps, the use of multiple retrieval methods, and the validation on evaluated suitable studies that will boost confidence by the researchers that all relevant studies are captured. The structured iterative manner also facilitates a better ability to trace-back decisions for re-evaluation in light of new discoveries and adjust when or where necessary.

In this paper, we assess the usability and performance of PALETTE on two literature reviews in PC. Furthermore, with a detailed description, we provide guidance on how to apply PALETTE for literature reviews in PC.

## Methods

In this section, we describe the phases of PALETTE and present the criteria for observation to provide insight into our initial experiences with the framework.

### Palette

**Fig. 1:**
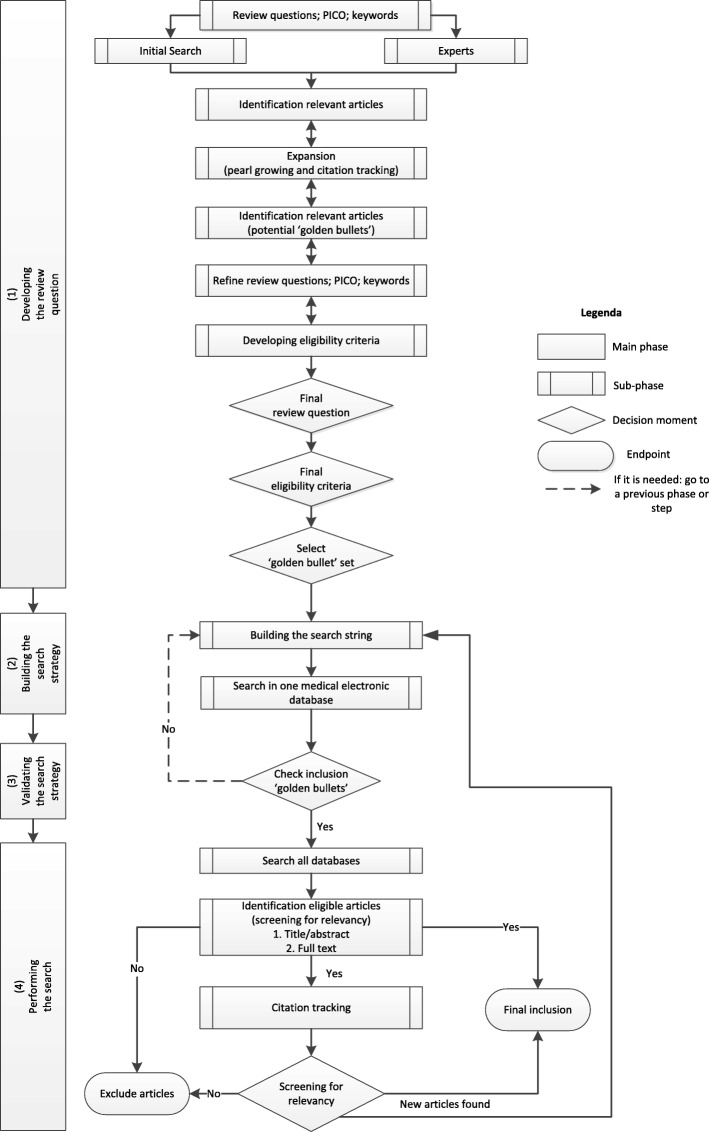
PALETTE: an iterative method for the search of a literature review

The iterative literature search, PALETTE, consists of four phases: (1) developing the review question, (2) building the search strategy, (3) validating the search strategy, and (4) performing the search. Each subsequent phase consists of sub-phases and is informed by what is previously learnt. Results from one phase could require the researcher to return to the previous phase. A detailed description of the phases, moments of decision-making, and techniques used is presented below and visualised in Fig. 1.

#### (1) Developing the review question

At the beginning of a PC review, the researchers first explore the key elements of the question carefully by performing an initial literature search. This search will be explorative, covering (a combination of) various topics from the initial review question supplemented with searches for reviews and overview articles to enhance the understanding of the overall perception within the field. In addition to the initial search, experts in the domain of interest are contacted to provide valuable articles. When experts cannot be contacted, it can be helpful to scan publications by key authors within a field to identify key papers and find relevant phraseology. Moreover, to overcome bias in the article set and to increase the body of knowledge, the key articles from the initial search and experts are expanded by adapted pearl growing (Table 1) and by both forward and backward citation tracking (Table 1).

After having collected all the references from the initial search, experts, and expansion, the researchers discuss the found body of evidence and map it to the initial review question whereby all related concepts are envisioned. When necessary, they refine, based on the added knowledge about the topic of interest, the review question, or concepts and thus the search strategy. This fine-tuning of the review question helps to address the most important viewpoints on the topic and, therefore, ensures a rich evidence-base. Furthermore, clear eligibility criteria are developed. Based on the final review question and the eligibility criteria, the researchers, preferably two researchers to minimise subjectivity, will select those articles from the retrieved articles that are relevant to the review question and fit into the eligibility criteria. These articles are the so-called ‘golden bullets’ and will be used for both fine-tuning the search query as well as the validation of the searches (Table 1).

This iterative process of screening the articles, fine-tuning the review question, modifying and developing the search strategy, and defining the eligibility criteria for answering the review question should be carefully explored by the researchers. It is of utmost importance that this process is well documented so that decisions leading to the final review question, the eligibility criteria, and the ‘golden bullets’ are transparent for the reader.

#### (2) Building the search strategy

The ‘golden bullets’ are analysed using PubMed PubReMiner (an online software tool that performs a frequency analysis of text words, MeSH terms, etc. on returned results from a PubMed query, Table 1), swift review (a programme to search, categorise, and visualise patterns in literature search results, Table 1), and manual identification of frequently occurring terms, phrases, index keywords and concepts. This input is used to compose a search query and this search is run in the most appropriate medical electronic database for the topic.

#### (3) Validating the search strategy

To validate the search strategy built in phase 2, the researchers check whether all ‘golden bullets’ can be identified within the results of the new literature search. If not, the literature search must be adjusted and the process of searching should be repeated. For certain topics, a search query might even be composed of several parallel queries, a so-called multithreaded search query. Since concepts within the corpus are so dispersed, the only way to capture all references is to construct several queries consisting of different combinations of concepts which are run in parallel to reach optimal retrieval. When all ‘golden bullets’ are identified, the researchers can continue to the next phase of PALETTE with the built search strategy.

#### (4) Performing the search

The researchers adapted the final search strategy developed in the second phase of PALETTE to other relevant electronic databases and run the search in these databases. This is followed by screening and selection of the articles using the predefined eligibility criteria. The choice of additional databases depends on the topic, journals covered in the database, and the likelihood of containing relevant information. The resulting articles from this step will be included in the review. As a final check of completeness, both backward and forward citation tracking will be performed for potentially missed relevant studies (Table 1). Citation tracking aims to identify new potentially eligible studies and to determine whether highly specific and relevant terminology was missed. If so, the search query should be adapted. Based on the missed articles, the keywords must be adjusted, the literature search in all electronic databases must be repeated, relevant articles should be identified, and citation tracking must be performed (this step could be repeated several times). When no new articles are qualified for inclusion, the final set of relevant articles is reached and the iterative process is completed.

### Criteria to evaluate PALETTE

Our research team has recently performed two literature reviews in PC, which offers the opportunity to present practical experiences with applying PALETTE. The first review involved healthcare professionals’ experiences in providing Paediatric Palliative Care (LR1). The second review concerned patients’ experiences with Advance Care Planning (ACP) (LR2).

Firstly, we share user experiences to elicit relevant aspects of the process of PALETTE: use of experts, development of the review question, and understanding of the topic of interest. Secondly, the value of PALETTE was evaluated by comparing the PALETTE results for both LR1 and LR2 with results retrieved from a recommended search method for reviews of intervention studies (PICO). Criteria were number and value of identified ‘golden bullets’, NNS, and comprehensiveness of the search.

## Results

### Developing the review question

The input of experts in the phase of developing the review question was only applied in LR2 (Table 2). Thirty-three experts, identified as persons who were actively involved in ACP research and/or practice and, as such, were familiar with ACP literature, were asked to recommend relevant articles regarding the review question. This resulted in six potentially relevant articles. Although these six articles were helpful in fine-tuning the focus of the study, after close inspection and discussion within the research team, none of them became part of the ‘golden bullets’.

**Table 2:** Description of initial observations and user experiences during the application of PALETTE

Topic	Experience LR1		Experience LR2	
Developing the review question: Initial review question	‘What are experienced barriers in practicing Paediatric Palliative Care from the perspective of professional caregivers?’		‘How do patients experience and respond to ACP in palliative care?’	
Developing the review question: experts	Approached: 0 experts.		Approached: 33 experts. Result: 6 potentially relevant studies of which none became a ‘golden bullet’.	
Developing the review question/validation: ‘golden bullets’	33 ‘golden bullets’ were identified		7 ‘golden bullets’ were identified.	
Developing the review question: Adjusted review question	What barriers and facilitators in providing Paediatric Palliative Care are experienced by healthcare professionals?’		Not applicable	
Developing the review question: PICO/keywords	‘Barrier’, ‘facilitator’ and, ‘need’ were removed from the search strategy.		The method of data collection was added to the search strategy.	
Final review question	‘What are the experiences of healthcare professionals when providing Paediatric Palliative Care?		’What are the experiences with ACP of patients with a life threatening or life-limiting illness?’	
Performing the search: number to screen	*Traditional search (Medline):* • 2815 articles • 31 relevant articles	*PALETTE* *(Medline):* • 2600 articles • 42 relevant articles	*Traditional search I (Medline):* • 14,746 articles • 20 relevant articles Traditional search II *(Medline)*: • 5153 articles • 14 relevant articles	*PALETTE* *(Medline):* • 3555 articles • 20 relevant articles

*ACP* Advance Care Planning, *LR1* Literature Review 1, *LR2* Literature Review 2

The articles identified in this phase, were valuable for the research team in tuning between the information needed and the available information. Based on these articles in both LRs, the research question was refined, keywords were adapted and/or sharpened, and eligibility criteria were developed and tightened (Table 2).

### Building the search strategy

The identified ‘golden bullets’ of both LRs, were analysed both manually as well as with the use of software to identify frequently occurring terms, phrases, index keywords, and concepts. These words were subsequently used to build the search strategy in both LRs. This analysis appeared to be helpful for improving the search string, particularly to search more in-depth, which resulted in a more focussed search for both LRs.

### Validating the search strategy

For both LRs, not all ‘golden bullets’ could be identified in the results of the first search. Therefore, the reviewers returned to the previous phase and adjusted the search strategy. Once the ‘golden bullets’ were identified with the built search strategy and, consequently, the validation test was completed, the reviewers felt more certain that the final included articles represented a comprehensive set that covered the topic of interest.

### Performing the search

In comparison with the recommended search method for reviews of intervention studies (PICO), the NNS when applying PALETTE decreased in both LRs, whereas the number of relevant articles increased (Table 2). In LR1, the NNS decreased from 2815 (recommended search method) to 2600 (PALETTE) articles. At the same time, the number of relevant articles increased from 30 (recommended search method) to 42 (PALETTE). In LR2, the NNS decreased from 14,746 (recommended search method) to 3550 (PALETTE) articles, and included the 20 studies that were identified by PALETTE. As a common step in the recommended search method, the search was developed further, resulting in 5153 NNS. Where the NNS had decreased, the number of relevant articles also decreased. Six relevant articles were missed out of the 20 relevant articles identified applying PALETTE.

## Discussion

Constructing relevant, focussed review questions in PC is a daunting task and requires an intricate knowledge of this field and all its actors. The same applies to the terminology used and the ability to identify all relevant studies. To address these issues and the shortcomings of the current literature review methodology, mainly developed for intervention studies, we present PALETTE as a pragmatic framework, which encompasses multiple retrieval methods applied in an iterative transparent way. Although the different techniques used within PALETTE have been around for some time, we provide a framework to use these in a transparent and coherent way with a clear decisional tree. As such, we provide guidance for researchers in the field of PC as well as in other specialties challenged by explorative questions, heterogeneity, and poorly defined keywords and concepts when conducting a review. Not every single technique will lead to a proportional number of relevant articles in every review; however, using PALETTE ensures a high likelihood of retrieving relevant articles with confidence.

The introduced iterative method results in four main positive aspects. Firstly, because of the more qualitative nature and the poorly defined concepts, review questions in PC need preliminary exploration. If not, researchers run the risk of missing a related concept not envisioned at the beginning. When applying the more iterative approaches such as berry picking and pearl growing solely, [24, 25] it is difficult to maintain transparency concerning relevant article identification and introduces the possibility of bias. By having a clear framework, such as PALETTE with the precise reporting of each step, we overcome this problem and provide the researchers with an opportunity to evaluate the process. This is in line with the PRISMA (Preferred Reporting Items for Systematic Reviews and Meta-Analyses) guidelines, which underline the importance of transparent reporting [26]. In addition, the PRISMA flowchart can be complementary to PALETTE. To illustrate, once the final search string has been developed, the steps in PALETTE (phase 4) are comparable with PRISMA and can be reported according to the PRISMA flowchart.

Secondly, as compared with the search building methods in intervention studies, PALETTE enables the research team to provide input on opinions and views, which in-turn enables them to explain what works for whom, in what contexts, and why in a transparent manner [27, 28]. This is necessary for an in-depth understanding of the content of the topic in the still poorly defined field of PC [29].

Thirdly, the total body of evidence in an article on PC is not well captured in terms. Therefore, validation is required on an article level. By checking the ‘golden bullets’, PALETTE grants this opportunity and validates the literature search on content and not just on the presence of keywords. This technique within PALETTE results in a representative set of articles.

Lastly, PALETTE might offer greater proportionality between the efforts of the researchers and the results of the literature search. When using a Boolean logic search query based on the initial review question and using every conceivable terminology on its own, some of which are quite ambiguous, huge amounts of results (10s of thousands) have to be screened manually and highly relevant citations are still missed [30]. The literature search in PALETTE is guided by the keywords and the content of studies that undoubtedly should be part of the review (‘golden bullets’) to find an optimal balance between specificity and sensitivity to keep the NNS manageable. This became apparent in the comparison between the recommended search method for reviews of intervention studies and PALETTE for LR1 and LR2 in which the NNS decreased for both LRs whereas the number of relevant articles increased with the application of PALETTE. Additionally, the kind of evidence researchers are often looking for when performing a review in PC aims to discover the variety of experiences or all opinions. Therefore, it is less critical in comparison with studies about a specific intervention when not all studies are identified. A view does not necessarily gain importance with the number of studies found [22].

Four limitations of PALETTE should be considered when applying PALETTE. Firstly, regular feedback within the research team is necessary to fine-tune the review question and to keep focussed on the aim of the review. Secondly, care should be taken when compiling the ‘golden bullets’. The ‘golden bullets’ should reflect the topic well from multiple angles so as to not introduce a skewed data set. By combining wisely chosen experts with the initial literature search and the expansion of articles, the risk of a skewed data set can be avoided. Thirdly, the benefit of the involvement of experts was limited in our examples. In the literature, different opinions regarding the involvement of experts are evident [22, 29]. We argue that although time-consuming, the involvement of experts should remain a component of PALETTE. Especially because the involvement of experts could be valuable due to the experts’ intricate knowledge of their topic and their ability to identify key articles (potential ‘golden bullets’). The value of the involvement of experts could however depend on the content of the review. Finally, to ensure the quality of the iterative literature search, researchers should preferably collaborate with an information specialist. In such a collaboration, researchers can provide the information and specialist experience of clinical practice to explain concepts whereas the information specialist can contribute to the literature search with his/her knowledge about the most optimal way of retrieving data from the sources, including which software to use to optimise the literature search (Table 1). Therefore, the collaboration provides the ultimate opportunity to combine knowledge of practice and knowledge of software and techniques used during the literature search, as also stated by Beverly et al. [31].

Some strengths and limitations should be taken into account. PALETTE is a new approach that can be helpful in performing literature reviews in PC. However, we still have limited experience with the application of PALETTE and compared minimal results between PALETTE and the recommended search method. We, for instance, did not measure the costs in terms of time needed for each phase of PALETTE. Regarding the time needed, we know from previous research that an experienced reviewer can screen an average of two abstracts per minute, but abstracts for complex topics may take several minutes each to evaluate [32]. Given the decrease of NNS when using PALETTE, we hypothesise, that a significant amount of time will be saved in the sub-phase of ‘identification eligible articles’. Knowing these strengths and limitations of this study, we encourage researchers to use PALETTE and to evaluate the time needed for and the value of this method.

## Conclusions

We presented PALETTE, a transparent and coherent pragmatic framework to overcome the challenges of conducting a literature search for a review in PC. This guidance enables the researchers in a relatively young and developing specialty to maximise both sensitivity and precision in their search process. PALETTE helps to improve question development and increase the understanding of the topic of interest and the development of a literature search. Compared with the recommended search method, PALETTE provided greater balance between the NNS and identified relevant articles. Whilst our initial results with PALETTE are promising, more research would provide valuable data about the applicability of PALETTE within the field of PC.

## Abbreviations

ACP: Advance Care Planning
LR: Literature review
NNS: Number needed-to-screen
PALETTE: Palliative cAre Literature rEview iTeraTive mEthod
PC: Palliative care
